# Bacterial transcriptome reorganization in thermal adaptive evolution

**DOI:** 10.1186/s12864-015-1999-x

**Published:** 2015-10-16

**Authors:** Bei-Wen Ying, Yuki Matsumoto, Kazuki Kitahara, Shingo Suzuki, Naoaki Ono, Chikara Furusawa, Toshihiko Kishimoto, Tetsuya Yomo

**Affiliations:** Faculty of Life and Environmental Sciences, University of Tsukuba, Ibaraki, 305-8572 Japan; Graduate School of Information Science and Technology, Osaka University, Osaka, 565-0871 Japan; QBiC, RIKEN, Osaka, 565-0874 Japan; Graduate School of Information Science, Nara Institute of Science and Technology, Nara, 630-0192 Japan; Faculty of Science, Toho University, Chiba, 274-8510 Japan; Graduate School of Frontier Biosciences, Osaka University, Osaka, 565-0871 Japan; Exploratory Research for Advanced Technology, Japan Science and Technology Agency, Tokyo, 102-0076 Japan; Present address: IMS, RIKEN, Kanagawa, 230-0045 Japan

**Keywords:** Transcriptome, Experimental evolution, Heat shock, Thermal adaptation

## Abstract

**Background:**

Evolution optimizes a living system at both the genome and transcriptome levels. Few studies have investigated transcriptome evolution, whereas many studies have explored genome evolution in experimentally evolved cells. However, a comprehensive understanding of evolutionary mechanisms requires knowledge of how evolution shapes gene expression. Here, we analyzed *Escherichia coli* strains acquired during long-term thermal adaptive evolution.

**Results:**

Evolved and ancestor *Escherichia coli* cells were exponentially grown under normal and high temperatures for subsequent transcriptome analysis. We found that both the ancestor and evolved cells had comparable magnitudes of transcriptional change in response to heat shock, although the evolutionary progression of their expression patterns during exponential growth was different at either normal or high temperatures. We also identified inverse transcriptional changes that were mediated by differences in growth temperatures and genotypes, as well as negative epistasis between genotype—and heat shock-induced transcriptional changes. Principal component analysis revealed that transcriptome evolution neither approached the responsive state at the high temperature nor returned to the steady state at the regular temperature. We propose that the molecular mechanisms of thermal adaptive evolution involve the optimization of steady-state transcriptomes at high temperatures without disturbing the heat shock response.

**Conclusions:**

Our results suggest that transcriptome evolution works to maintain steady-state gene expression during constrained differentiation at various evolutionary stages, while also maintaining responsiveness to environmental stimuli and transcriptome homeostasis.

**Electronic supplementary material:**

The online version of this article (doi:10.1186/s12864-015-1999-x) contains supplementary material, which is available to authorized users.

## Background

Evolutionary experimentation is a powerful tool for the exploration of how living organisms adapt to environmental change and is commonly applied in studies of evolution, typically focusing on changes in genome sequences. The results of these studies have provided experimental evidence to substantiate or revise numerous theories of evolution [1, 2]; the evolutionary mechanisms were generally explained by changes in DNA sequences and/or some particular genes [3–6].

Nevertheless, adaptive evolution also works to optimize cellular gene expression; thus, transcriptome evolution is a relevant area of study. In addition to studies analyzing the transcriptome response to stimuli such as heat shock and nutritional stress [7–11], recent studies report the effects of highly abstract phenomena on global transcriptome parameters. For example, studies showing gene transcriptional changes that were correlated with growth rates, performed in both yeast [12–15] and bacteria [16–18], as well as studies of the trade-off relationship for gene expression responsible for growth fitness and the stress response [18, 19]. Our previous studies identified transcriptome-wide growth-induced transcriptome reorganization [18] and demonstrated that a similar global coordination was also found for stochastic adaptation independent of regulatory mechanisms [20].

Global optimization of gene expression is critical for living cells to achieve novel adaptive states in new environments and must occur not only during transient adaptive responses but also during long-term adaptive evolution [19]. The pioneering transcriptome studies using experimental evolution provided detailed transcriptional changes for genes that are specifically involved in evolution [21, 22]. Studies of parallel evolution in different environments usually found transcriptome diversity and a correlation to genetic background [23–25]. Although a number of studies have reported the general features of transcriptome evolution [6, 23–27], it is unclear whether transcriptome evolution requires alteration of the genomic response to acquire an adaptive state.

As a pilot investigation, we compared transcriptomes of the heat shock response and thermal adaptation. We previously performed experiments in thermal adaptive evolution with a laboratory *Escherichia coli* strain. We clarified the mechanism of genome evolution, *i.e.*, positive selection *vs.* neutral evolution [5]; however, how transcriptomes reorganize during the evolution of thermal adaptation remained unknown. The heat shock response is a critical mechanism that living cells use to tolerate high temperatures/thermal stress [28, 29]. In *E. coli*, this mechanism is largely dependent on feedback regulation from the sigma factor 32 gene (*rpoH*) and heat shock proteins such as GroEL/ES and DnaK [29, 30]. It is intriguing to know whether *Escherichia coli* cells alter this responsive machinery to reach adaptive states, or maintain heat shock responsivity using other expression patterns during evolution.

To address this question, we performed a microarray analysis with the representative ancestor (Anc) *Escherichia coli* cells, and three evolved cell populations (41B, 43B and 45 L), which were acquired at varied evolutionary periods and were well adapted to growth temperatures of 41.2, 43.2 and 44.8 °C, respectively. To distinguish the thermal stress response states and the states of adaptation to high temperatures, we obtained the transcriptomes of both the exponential growth phase at the different growth temperatures (designated as the steady states) and the heat shock response (designated as the responsive states). Multilevel analyses were conducted to investigate the evolution of thermal adaptive transcriptomes. Overall, our results found that long-term evolutionary adaptation to high temperatures was different for the transient response to thermal stress. We summarized these results by proposing a potential mechanism that illustrates transcriptome evolution accompanied by genome evolution.

## Methods

### Strains

**Fig. 1:**
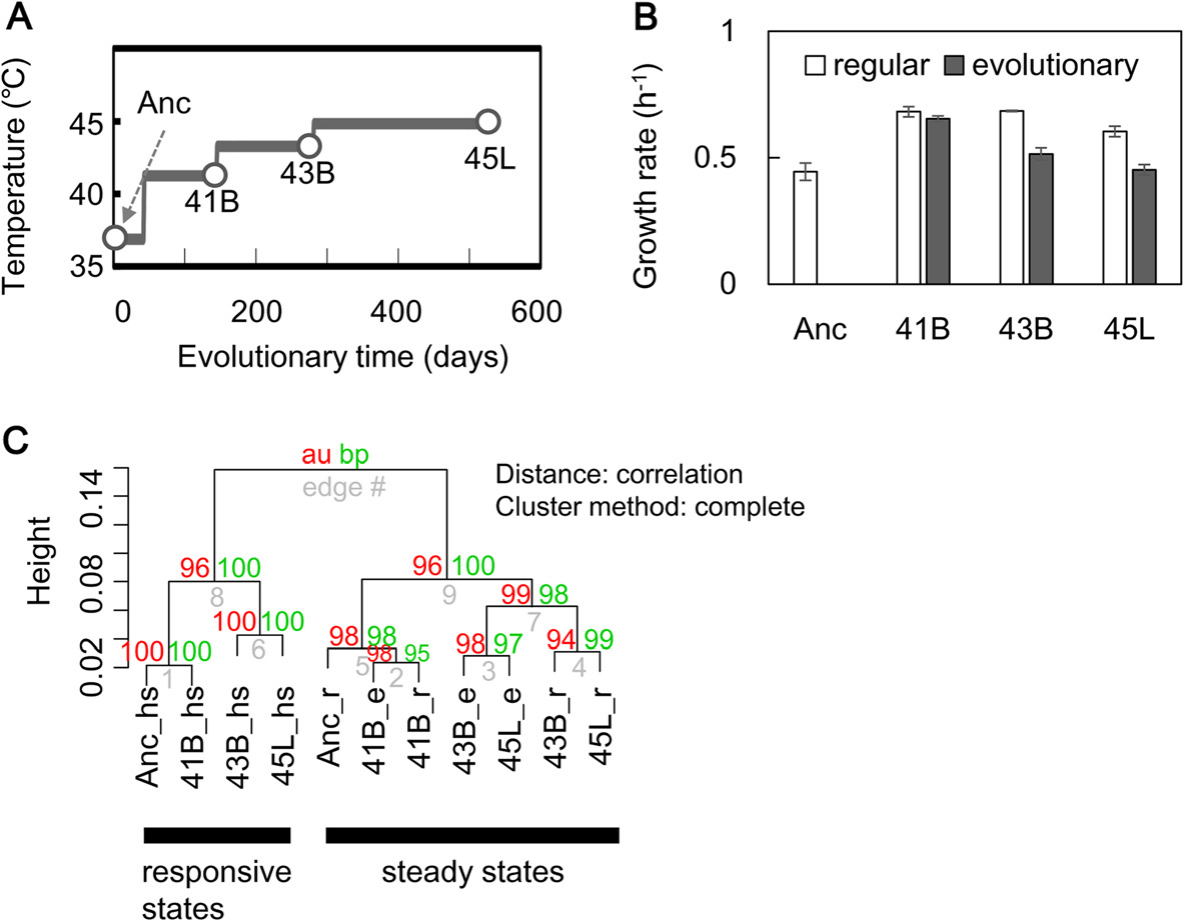
Gene expression of the cells experienced thermal adaptive evolution. **a** Overview of thermal adaptive evolution. The ancestor (Anc) and evolved strains (41B, 43B, 45 L) used in the present study are indicated, according to our previous report [5]. **b** Growth rates of the ancestor and evolved strains. Exponential growth rates at both regular (37 °C) and evolution-stimulating (high) temperatures are shown as open and filled bars, respectively. Standard errors of three independent tests are indicated. **c** Dendrogram of gene expression. Gene expression patterns are clustered as indicated. Mean of the repeated microarray results is used. The three experimental conditions of heat shock, exponential growth at regular and evolutionary temperatures are indicated as hs, r and e, respectively. Black bars highlight the two categories of gene expression, designated as responsive and steady states, respectively

The *Escherichia coli* strains analyzed in the present study were from a thermal adaptive evolution study performed previously [5]. The genetically engineered *Escherichia coli* strain *DH1ΔleuB::gfpuv5-Km*^*R*^ was used for the evolution experiment, in which the cells were serial transferred with a temperature upshift, from 36.9 to 44.8 °C. The ancestor and evolved strains were designated as Anc, 41B, 43B and 45 L, respectively (Fig. 1a). The genome mutations were determined as previously reported [5]. These four cell populations were subjected to the microarray analysis.

### Cell culture and growth

The *Escherichia coli* cells were cultured in 5 mL of minimal medium M63, supplemented with 2 mM leucine and 25 μg/mL kanamycin, at corresponding temperatures. The culture conditions were exactly the same as previously reported [5]. Cells were counted using flow cytometers, either the FC500 (Beckman) or the FACSCanto II (Becton-Dickson). Cell concentrations were calculated as the ratio of gated particles representing the number of *Escherichia coli* cells carrying the reporter gene *gfp* (green fluorescent protein) and beads of known concentrations, as previously described [18, 31]. The growth rates were calculated using the initial and final cell concentrations and the culturing time, as described elsewhere [18, 20, 31]. The initial and final cell densities were maintained at approximately 1 × 10^4^ and 2 × 10^8^ cells/ml, respectively. The culturing times were varied from 14 to 23 h. Cells within the exponential growth phase were collected for microarray analysis.

### Heat shock experiments

The condition for the heat shock experiment was a 5-min incubation following a temperature upshift from 36.9 °C to 44.8 °C, as previously described [11]. Exponentially growing cells at approximately 2 × 10^8^ cells/mL were rapidly transferred to an adjacent water bath incubator (Personal-11EX; Taitec) set at 44.8 °C. Following a 5-min incubation at 44.8 °C, the cell culture was immediately poured into a cold phenol-ethanol solution to prepare the samples for microarray analysis. Each heat shock experiment was performed separately to enable precise control of the timing of the heat shock to ensure that the mRNA levels accurately reflected the stress response.

### Sample preparation and microarray

Three independent cell cultures were used for the microarray analysis to acquire the mean expression under each culture condition. RNA sample preparation, microarray analysis with the Affymetrix GeneChip system, and data extraction were all based on the finite hybridization (FH) model [32, 33] and were performed as previously described [18, 20]. A single-nucleotide tilling array (high-density DNA microarray) that covers the entire *Escherichia coli* genome [34] was used. Thus, single-nucleotide substitution bias within ORF (open reading frame) regions could be disregarded in our evaluation of gene expression level. The results of 32 arrays for four bacterial strains in steady and responsive states were used for the analysis, which included triplicates of exponential growth at regular (36.9 °C) and evolution-stimulating (41.2, 43.2 or 44.8 °C) temperatures and duplicates of heat shock conditions.

### Data normalization and annotations

Expression data sets from biological replicates were shown as mean values. The raw data sets were subjected to global normalization, resulting in a common median value of zero (logarithmic value) in all data sets, as previously described [18]. The data sets of a total of 4383 genes were used in the analysis. Both the normalized expression data sets and the raw CEL files were deposited into the NCBI Gene Expression Omnibus database under the GEO Series accession number GSE61749. The full datasets of gene names, gene categories [35], and gene regulations (TF) were downloaded from GenoBase, Japan (https://dbarchive.biosciencedbc.jp/jp/genobase-v6/desc.html) and RegulonDB v8.0 [36] (http://regulondb.ccg.unam.mx), as described previously [11, 18].

### Computational analysis

Binomial tests were performed to evaluate the statistical significance of extracted gene groups. These statistical analyses were carried out using free software packages available from the Broad Institute (http://www.broadinstitute.org). All statistical tests and computational analyses, except for the gene set enrichment analysis (GSEA), were performed using R [37]. Gene sets enrichment analysis (GSEA) [38] was performed as previously described [11, 18]. The gene categories and gene regulatory links (TF) comprising more than 15 genes were used for the enrichment analysis and bimodal tests. Principal component analysis (PCA) [39, 40], which classifies expression patterns according to gene expression level variance between the culturing conditions, was performed as described previously [18, 20].

## Results and discussion

### Overview of transcriptome evolution for thermal adaptation

To identify changes in gene expression for thermal adaptation, four *Escherichia coli* strains were acquired during experimental evolution that we performed previously [5]. We used an ancestor (Anc) population and evolved cell populations 41B, 43B and 45 L, which were adapted to the temperatures 41.2, 43.2 and 44.8 °C, respectively (Fig. 1a). Their growth rates were examined at both the regular (_r) and evolutionary high (_e) temperatures. The results showed that the evolved cells demonstrated highly recovered growth fitness at their corresponding evolutionary temperatures (Fig. 1b), consistent with our previous report [5]. These growing cells were collected for microarray analysis, such that growth rates corresponded to transcriptomes at the exponential growing phases analyzed in this study (Additional file 1: Figure S1). In addition, we acquired the transcriptomes of cells undergoing a heat shock response (_hs, a transient responsive state induced by a temperature increase from 36.9 to 44.8 °C) to identify whether thermal adaptation changed responsivity to a temperature increase (Additional file 1: Figure S1).

An overview dendrogram based on hierarchical clustering analysis clearly shows that global gene expression patterns could be divided into two main clusters, the responsive and the steady states, which corresponded to the transcriptomes during heat shock response and the exponential growing phases, respectively (Fig. 1c). These results suggest that the transcriptome reorganization that occurs during thermal adaptive evolution differs from the reorganization that occurs during a heat shock response. In addition, the transcriptomes of cells in the early and the late evolutionary processes were roughly separated, within the steady-state cluster. This tendency suggests that genetic changes (*i.e.*, genome mutations) played a role in the transcriptional changes during the evolutionary process at 43.2 °C (from 41B to 43B). According to the genome sequence analysis performed previously [5], a comparable number of genome mutations responsible for transcriptional regulation were newly accumulated in each strain (summarized in Additional file 1: Table S1). Overall, these results suggest that global transcriptome optimization might underlie transcriptome evolution.

### Equivalent magnitudes of responsive changes

**Fig. 2:**
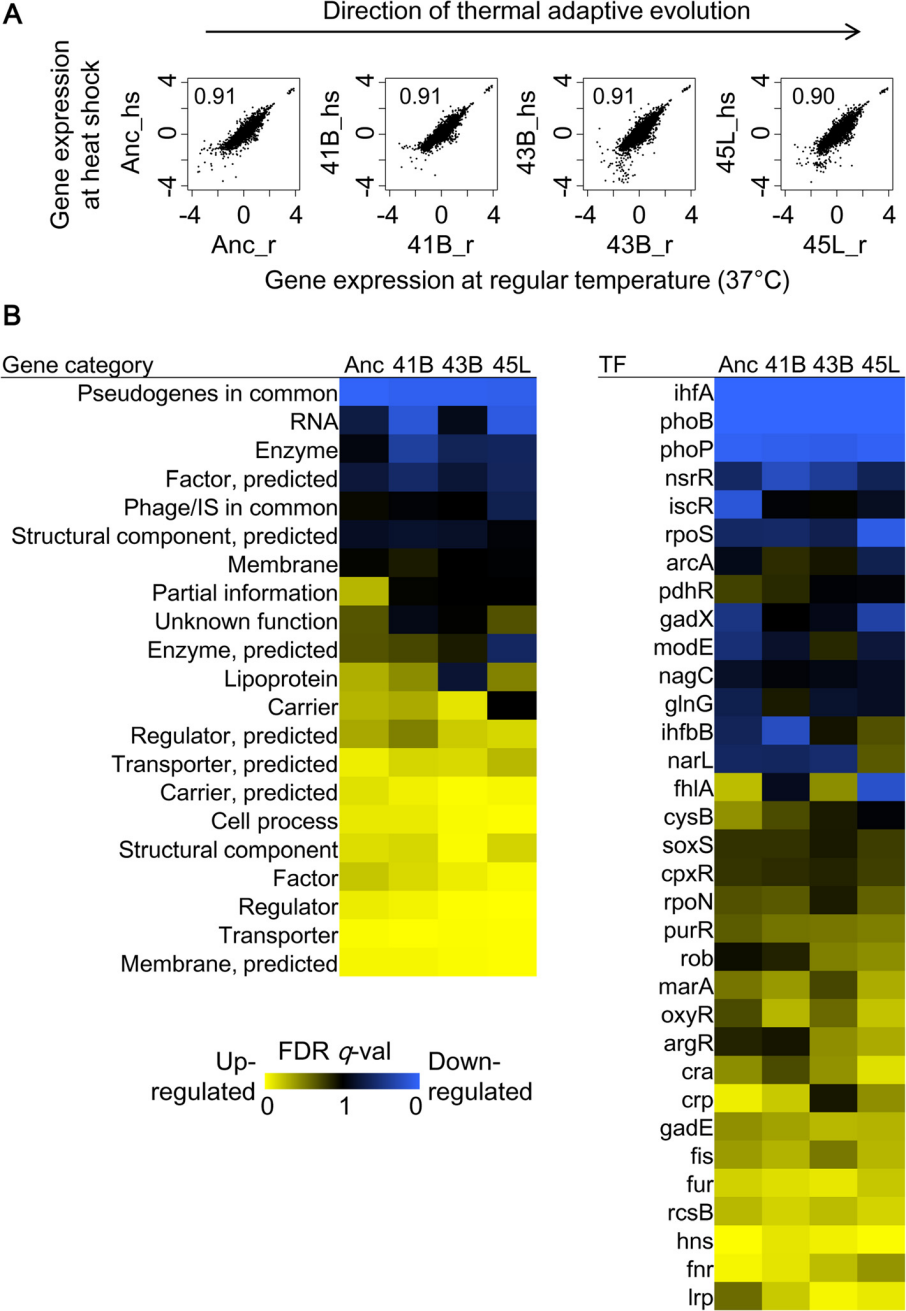
Changes in gene expression at heat shock responsive states. **a.** Scatter plots of gene expression in response to heat shock. Individual steady-state expression at regular temperature (_r) is plotted against its heat shock expression (_hs). The strain names and their correlation coefficients are indicated. The direction of evolution is indicated with the arrowed line for reference. **b.** Heat maps of enriched gene groups showing differentiated expression. The results of GSEA based on two types of annotations, gene category and gene regulation (TF), are shown in the left and right panels, respectively. The statistical significance (FDR *q* value) is indicated in the heat map. Vivid colors represent high significance in the directions of either up-regulated (yellow) or down-regulated (blue) genes

Because the heat shock response is a common reaction to a transient increase in temperature in living cells, we evaluated whether thermal adaptation reduced the magnitude of the general transcriptional changes associated with the heat shock response. Comparison of gene expression during the regular temperature and at heat shock showed that, according to the correlation coefficients, heat shock transcriptional reorganization occurred in the evolved strains, with comparable magnitudes to that found in the ancestor strain (Fig. 2a), which was consistent with the hierarchical clustering results (Fig. 1c). This similarity in the response to heat shock was confirmed by the fact that the expression of the major heat shock genes showed equivalent induction in all strains (Additional file 1: Figure S2A). These results suggest that the evolved strains maintain responsivity and sensitivity to a temperature increase, regardless of long-term evolution under high temperatures.

The enrichment analysis consistently found similar patterns of transcriptional change in most gene categories among all strains, although the genes in the categories of partial information and carrier showed significant differences between Anc and 45 L (Fig. 2b, left). A similarity in the patterns of transcriptional change was also observed for transcriptional regulation. Despite the small number of regulatory networks identified, such as the gene networks regulated by *iscR* and *fhlA*, most gene regulation patterns maintained identical directional changes among all strains (Fig. 2b, right). We note that such comparable changes in gene expression were also found in the transcriptional networks controlled by the global regulators harboring nonsynonymous substitutions or deletion mutations (*e.g.*, *lrp* and *oxyR*; Additional file 1: Table S1). These results indicate that thermal adaptive evolution did not disturb the mechanism of heat shock response and that the evolved competence at high temperatures might have been acquired using other pathways.

### Differentiated transcriptomes at steady states

**Fig. 3:**
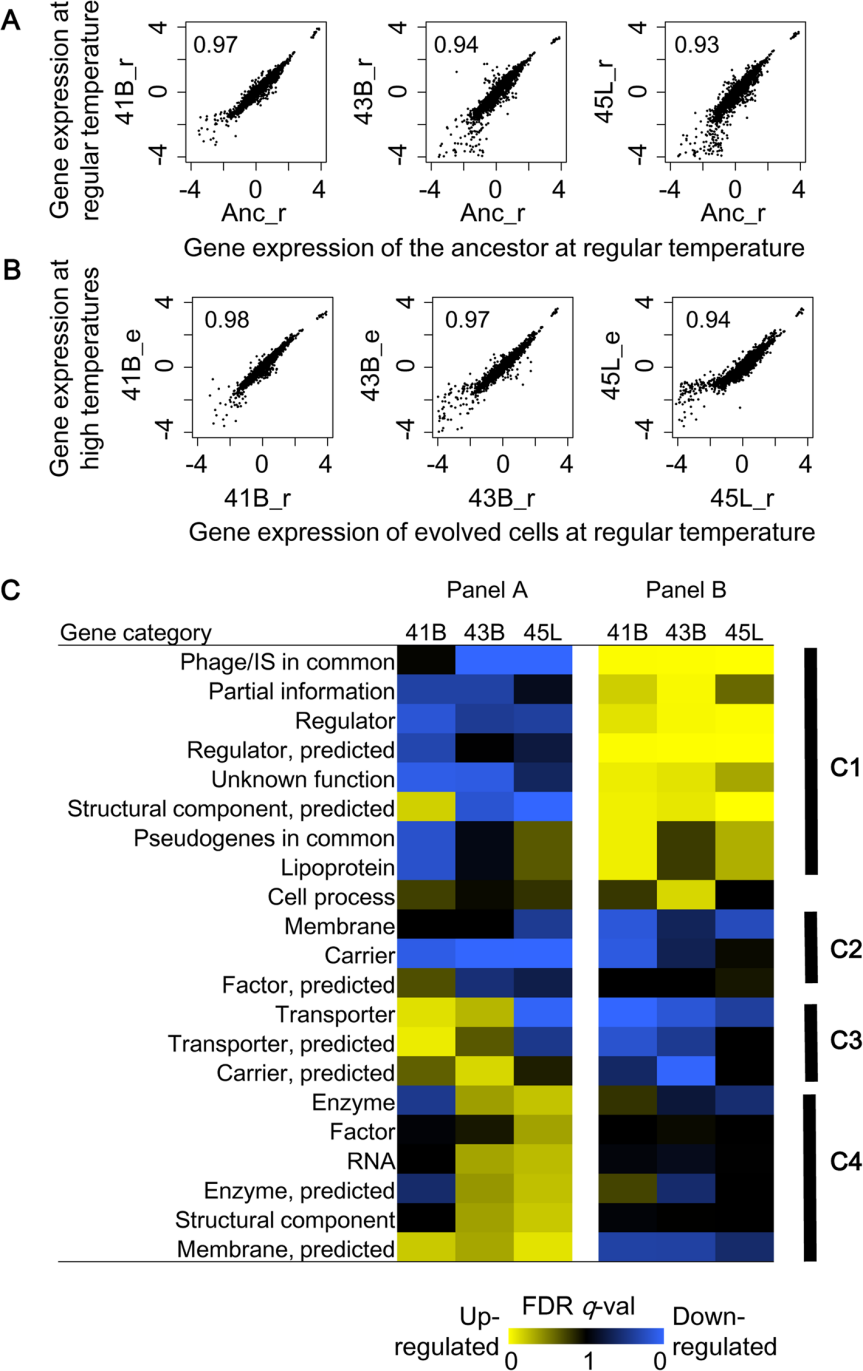
Changes in gene expression at steady states. **a** Scatter plots of gene expression at steady states under the regular temperature. The gene expression of the ancestor strain at regular temperature (Anc_r) is plotted against gene expression of the evolved strains at regular temperature (41B_r, 43B_r and 45L_r), which represents a comparison of genotypes (genome mutations). Correlation coefficients are indicated. **b** Scatter plots of gene expression at steady states under the evolution-stimulating temperatures. The gene expression patterns of the evolved strains at regular (41B_r, 43B_r and 45L_r) and evolutionary-stimulating (41B_e, 43B_e and 45L_e) temperatures are plotted individually, representing a comparison of the growth temperatures. Correlation coefficients are indicated. **c** Heat maps of enriched gene groups of differentiated expression. The results of GSEA based on gene category are shown. The left and right panels represent comparisons of genotypes and growth temperatures, respectively, corresponding to (**a**) and (**b**) panels, respectively. The statistical significance (FDR *q* value) is indicated in the heat map. Vivid colors represent high significance in the directions of either up-regulated (yellow) or down-regulated (blue) genes. The strains are indicated. The labeled black bars (C1–C4) located at the right of the heat maps indicate the manually categorized clusters showing distinguished changing patterns

Because thermal adaptive evolution did not alter the sensitivity of the transcriptome to heat shock, we subsequently analyzed whether and how thermal adaptive evolution disturbed the steady-state transcriptomes at regular and evolution-stimulating temperatures. Comparison of the ancestor and the evolved steady-state transcriptomes showed that longer evolution proceeded larger differentiation (*i.e.*, lower correlation) of the transcriptome from the ancestor at the regular temperature (Fig. 3a). These results suggest that thermal adaptive evolution optimized the global gene expression patterns, not only at evolution-stimulating high temperatures but also at the regular temperature, which did not simulate an evolutionary condition. These results are supported by the fact that the evolved cells all grew faster (improved growth fitness) than the ancestor strain at the regular temperature (Fig. 1b). In addition, comparison of the steady-state transcriptomes at the different growth temperatures showed that transcriptome reorganization for thermal adaptation corresponds with the evolutionary process; *i.e.*, the correlations of gene expression in the steady states at the regular and evolution-stimulating temperatures became weaker in the order of 41B, 43B and 45 L (Fig. 3b), in congruence with the evolutionary process that actually occurred (Fig. 1a).

Gene set enrichment analysis (GSEA) showed that transcriptome reorganization for thermal adaptation did not randomly occur but followed directional changes (Fig. 3c). The changes in gene expression compared in Fig. 3a represent the transcriptome differences derived from genotypes (*i.e.*, the genomes harboring all the mutations fixed during the thermal adaptive evolution) and similar differences in Fig. 3b are derived from the growth temperatures. Gene function enrichment analysis was performed on the two types of differences, the genotype- and the growth temperature-mediated changes. Roughly, the changes in gene expression mediated by the genotypes were inversely correlated to changes mediated by the growth temperatures (Fig. 3c). The resultant patterns could be manually divided into four clusters (C1–C4). C1 showed significant inverse correlations; C3 and C4 showed gradual directional changes. Such directional changes were also found in regulatory networks (Additional file 1: Figure S3). The inverse transcriptional changes reflected a homeostasis of the transcriptome, which is supported by the finding that the expression of genes responding to a temperature increase (*i.e.*, heat shock genes) returned to normal levels in all evolved cells, regardless of the high temperatures (Additional file 1: Figure S2B).

### Thermal adaptive transcriptomes between the regular and the heat shock states

**Fig. 4:**
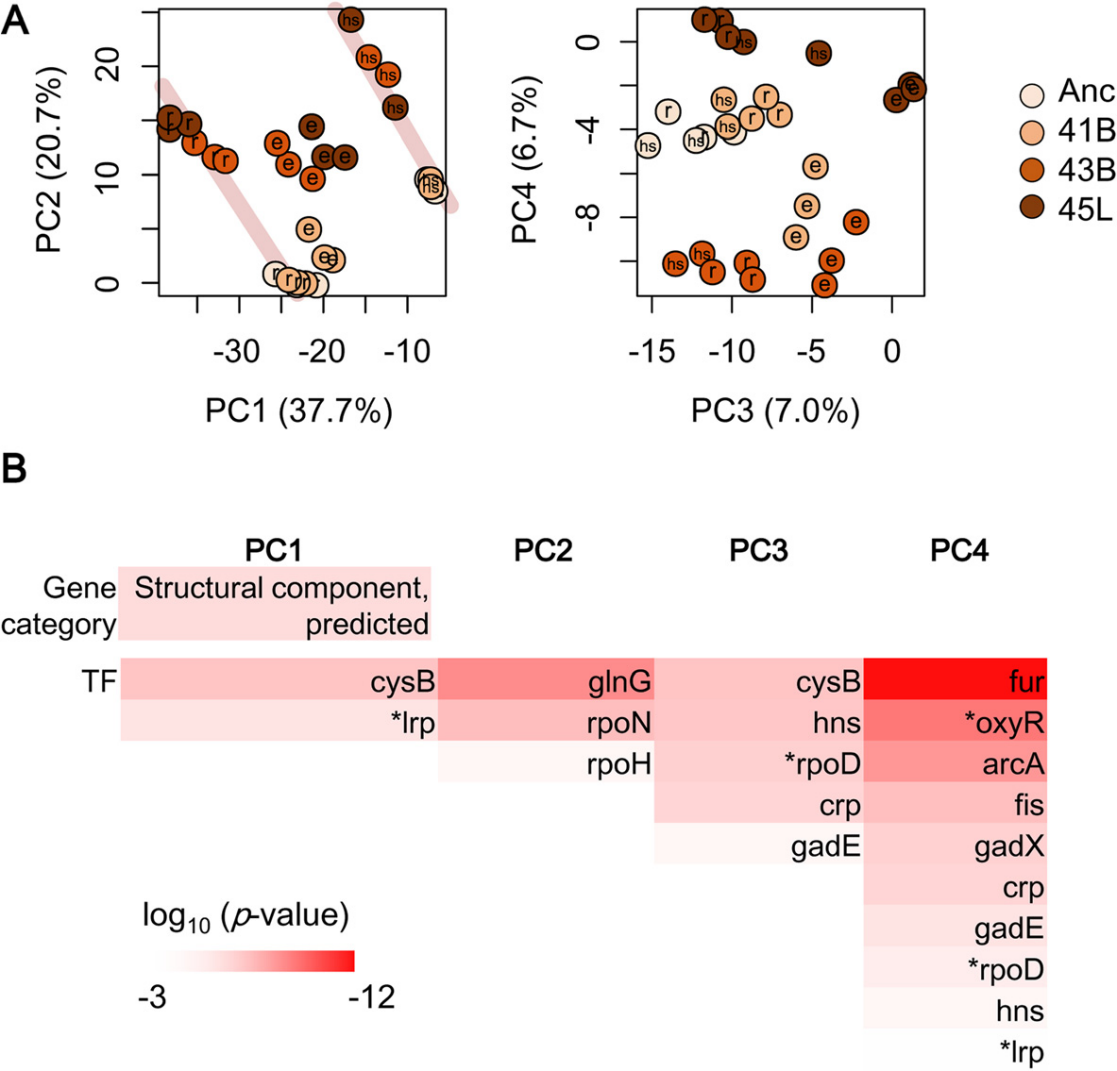
Transcriptome reorganization for thermal adaptation. **a** Principal component analysis based on individual microarrays. The main four components (P1–P4) are shown. Color variation filled in the circles represents differences among the strains (Anc, 41B, 43B and 45 L), as indicated. The letters hs, r, and e, which are indicated within the circles, represent gene expression conditions, heat shock responsive states, and steady states at regular and evolution-stimulating (high) temperatures, respectively. The weights of each PC are indicated. Pale gray and pink lines illustrate the two zones of the steady expression at the regular temperature and the responsive expression, respectively. **b** Gene categories and gene regulation significantly contributed to the main PCs. The top 5 % of the genes (439 genes) weighted on each PC were subjected to analysis. Color bars indicate the statistical significance in log-scaled *p* values obtained using binomial tests with Bonferroni corrections. Asterisks indicate either non-synonymous single-nucleotide substitutions or InDel mutations that occurred during evolution

To identify the evolutionary direction of the thermal adaptive transcriptome, principal component analysis (PCA) using all data sets was performed. Analysis based on either the individual data sets (Additional file 1: Figure S1B) or the averaged data sets (Additional file 1: Figure S1A) resulted in the same conclusions (Fig. 4, Additional file 1: Figure S4). Overall, transcriptome reorganization could be roughly interpreted in four main PCs (representing approximately 72 % and 85 % of the total variance in Fig. 4a and Additional file 1: Figure S4A, respectively). Although the magnitude of the changes in gene expression in response to heat shock was approximately consistent in all strains (Fig. 2), the PC1/PC2 space showed that the difference between the transcriptome at evolution-stimulating temperatures and that at the heat shock temperature became smaller when evolution proceeded (Fig. 4a, left). In particular, PC1 represented the adaptivity of the strains in the corresponding conditions because PC1 was correlated with the growth rates (Additional file 1: Figure S5), as previously reported [14–16, 18]. The thermal adaptive expression patterns were located between the steady expression at regular temperature and the responsive expression to heat shock (Fig. 4a, left). We assumed that the adaptive transcriptome was detached from the responsive states and approached the novel steady states. In addition, PC3 and PC4 were likely related to the evolutionary process and to variation in genotypes, respectively (Fig. 4a, right). As shown in Fig. 4a, 43B and 45 L were located at the opposite sides of the Anc–41B zone in PC4. These results suggest that the genetic changes were distinct for thermal adaptation in the later evolutionary phase.

Functional enrichment analysis was performed for the genes positively and negatively loaded on the four main PCs (top 5 % each, 439 genes in each PC). The enrichment gene analysis (*p* < 0.001) failed to identify any essential functions (Fig. 4b, Additional file 1: Figure S4). The result suggests that transcriptome adaptation is not simply achieved by a central function of gene activity but is largely mediated by genes with diverse functions. The enrichment of transcriptional networks analysis resulted in largely varied transcription factor regulatory links (TF, *p* < 0.001) (Fig. 4b). The results showed that only a few regulatory networks were enriched in the most weighted components, PC1 and PC2, indicating that transcriptional networks participated as hubs in the transcriptome, and considerably contributed to thermal adaptation. In comparison, more regulatory links were enriched in the lower weighted components, PC3 and PC4. Differentially expressed regulatory networks were most abundantly identified in PC4, which represented variation in genotypes. This is consistent with the fact that the mutated regulators were largely identified in PC4 and, furthermore, demonstrated that genome mutations (*lrp*, *rpoD* and *oxyR*) did contribute to transcriptional changes at regulatory levels. In addition, of the mutated genes, most were nonsynonymous or InDel (insertion or deletion) mutations, found at considerable frequencies in PC1 and PC4 (Additional file 1: Figure S6). These findings indicate that genome evolution shaped the transcriptomes at PC1, which correlates with growth direction, and at PC4, which correlates with genotype.

### Negative epistasis in transcriptome reorganization

**Fig. 5:**
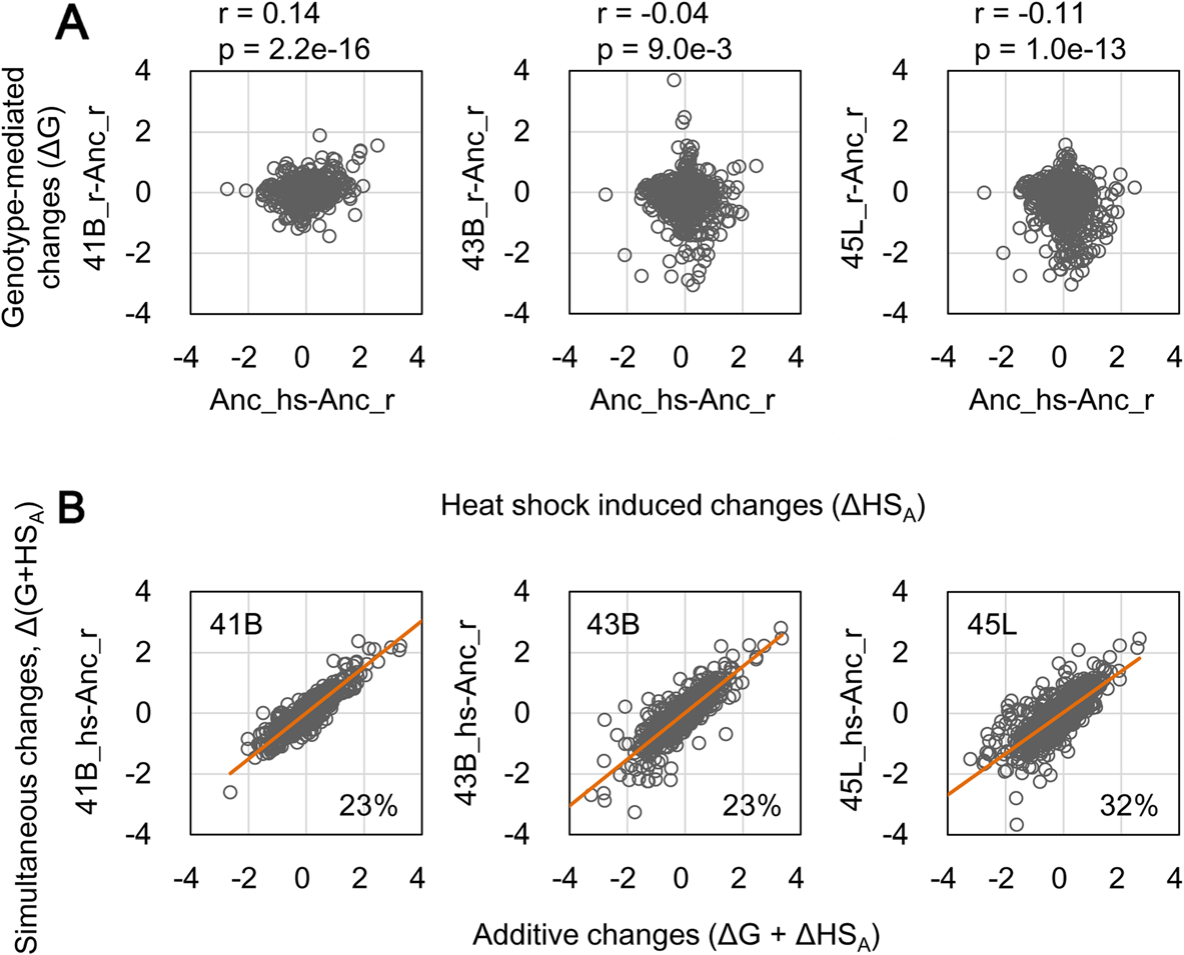
Epistasis in transcriptome between heat shock and genome mutation. **a** Scatter plots of the changes in gene expression mediated by heat shock and genotype. The heat shock-induced changes in gene expression (ΔHS_A_) are plotted against the genotype-mediated changes in gene expression (ΔG) at the regular temperature. Both changes are based on the ancestor (Anc). Correlation coefficients (r) and *p*-values (p) are indicated. **b** Negative epistasis. The additive changes in gene expression (ΔHS_A_ + ΔG) are plotted against the simultaneous changes (Δ(G + HS_A_)), which represent the changes in gene expression between the evolved strains at the heat shock temperature (41B_hs, 43B_hs and 45L_hs) and the ancestor under the regular temperature (Anc_r). The red line indicates linear regression. The values with slopes subtracted from 1 are indicated, representing the magnitudes of the negative epistasis. Gene expression is in log-scale

Considering our findings of inverse correlations (Fig. 3) and novel adaptive steady states (Fig. 4), we hypothesized that the contributions from genome mutations and temperature increase were negatively correlated. In other words, together, these factors cancel any effect on transcriptome reorganization. Because transcriptional changes caused by heat shock and genome reduction had a negative epistasis relationship [11], we next evaluated whether such a negative epistasis existed in the transcriptomes during thermal adaptive evolution. The transcriptional changes triggered by genome mutations that had accumulated from the ancestor strain (genotype-mediated changes, ΔG) showed low or no correlation to that induced by temperature increase in the ancestor strain (heat stock-induced change, ΔHS_A_) (Fig. 5a), which was different from a previous study that reported a significant positive correlation between genotype-mediated and heat shock-induced transcriptional changes [11]. As the process of evolution progressed, the weak correlation between the two contributors, genotype and heat shock, seemed to turn from positive to negative. These results indicated that the genome mutations and heat shock contributed to transcriptome reorganization nearly independently, although their interaction also had an effect on evolution.

Despite the weak correlations between genotype and heat shock, a negative epistasis in transcriptome reorganization was clearly detected (Fig. 5b). An approximate 20–30 % reduction in transcriptional changes was estimated to be the result of the simultaneous occurrence of genome mutations and heat shock, compared to their additive occurrence. This result is highly consistent with a previous finding that reported a ~30 % reduction in transcriptional changes [2]. The negative epistasis was most significant for genes that were highly weighted in the main PCs, *e.g.*, a ~50 % reduction in overlapping genes from PCs1–4 (Additional file 1: Table S2). This negative epistasis explains the fact that changes in gene expression were largely inversely related to genotype and temperature (Fig. 3c). Taken together, the changes in gene expression attributed to genetic alteration tended to cancel the changes attributed to the environmental perturbation. Such annulment might enable the cells to maintain the cellular homeostasis of the transcriptome. Although the underlying mechanism was not clearly resolved, our results suggest that adaptive evolution might involve a process of repressing gene expression changes triggered by multiple factors.

### A potential path of transcriptome evolution for thermal adaptive

**Fig. 6:**
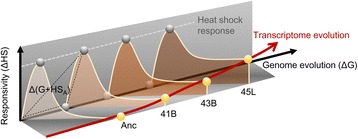
Schematic drawing of the proposed evolutionary trajectory. Changes in gene expression in response to a temperature increase are illustrated with transparent distributions, representing the well-known feedback regulatory mechanism in the heat shock response. The responsive states at heat shock are indicated with circles located at the peaks of the distributions indicated with the names of the ancestor and evolved cells. The equivalent heights of the distributions (*i.e.*, the vertical distances from the X-axis to the peaks) indicate that the heat shock response (ΔHS) of both the ancestor and the evolved strains remained in common. The steady states of the evolved cells at the regular temperature are shown with circles located on the axis for genome evolution and are shadowed by the transparent distributions. The order of these strains (*i.e.*, the distances from the starting point of the X-axis) represents the changes in gene expression (ΔG) mediated by the genotypes (*i.e.*, genome mutations). The steady states of the ancestor and evolved cells at the corresponding growth temperatures are highlighted with bright yellow circles. These steady states (circles) are placed at the sides opposite to the regular states along the distributions, suggesting that the thermal adaptive states were distinct to the regular steady states. In addition, they are set on the tails of the distributions, which implies that the steady states under high temperatures were relaxed from the heat shock stress to the corresponding thermal adaptive states. The broken triangle represents an example (41B) of the negative epistasis at transcriptome, as described in the main text. The solid red line, linking up the four steady states at the corresponding evolutionary temperatures, indicates the trajectory of transcriptome evolution along with genome evolution

Considering that transition from a heat shock state to a regular steady state generally explained the negative feedback mechanism [28, 29], which was regulated by sigma factor 32 (*rpoH*) and molecular chaperones, we proposed an evolutionary trajectory for thermal adaption of the transcriptome (Fig. 6). The cell population order corresponds to the evolutionary process (Fig. 1a). The responsivity (Y-axis, the gray circles at the peaks of the distributions) to a transient temperature increase was maintained normally, independent of long-term evolution (Fig. 2). Genome evolution (genome mutations) caused a differentiation of gene expression at the steady states in the order of genome evolution, even under the common regular temperature (X-axis, the hidden gray circles) (Fig. 3). The adaptive transcriptome transitions followed relaxation curves (the distributions in red and ivory), resulting from the feedback mechanism to the heat shock response. Normally, as the result of a heat shock response and negative epistasis (Fig. 5), thermal adaptive evolution of the transcriptome occurs as an extended relaxation process (Fig. 6, the red arrowed line), in which the steady states (Fig. 6, yellow circles) gradually return from the responsive states (Fig. 6, gray circles), bound for a relaxed state different from that of the steady states at the regular temperature (Fig. 6, hidden gray circles). This trajectory represents thermal adaptive evolution with a preference for fewer transcriptional changes between the transient and long-term temperature increase, which was verified by the increasing positive correlations, from 0.48 to 0.72 in 41B to 45 L, between the heat shock-induced changes in gene expression and the growth temperature-mediated changes in gene expression (Additional file 1: Figure S7). The resultant triangles (broken black lines, as an example for 41B) that formed as a result of the changes initiated by heat shock and genome mutations separately and simultaneously represent the cancellation effect on the transcriptome (negative epistasis, Fig. 5b), and this effect possibly became stronger during the course of evolution. We hypothesize that unknown global feedback mechanisms might have played an essential role in transcriptome evolution for thermal adaptation. In summary, thermal adaptive evolution is a process that not only drives the genome from positive to neutral evolution [5] but also optimizes the transcriptome for a balance between responsivity and adaptivity.

## Conclusions

Thermal adaptation in *Escherichia coli* cells differentiated steady-state transcriptomes under both normal and evolution-stimulating high temperatures; nevertheless, the heat shock response was maintained with a high sensitivity to thermal stress, as found in the ancestor population. Thermal adaptive evolution directed the transcriptomes to novel steady states different from regular steady states or responsive states. These findings indicate that long-term evolution does not alter existing response machineries but rather adjusts gene expression homeostasis to adaptive steady states.

## Availability of supporting data

The raw data is available at the GEO database under:

http://www.ncbi.nlm.nih.gov/geo/query/acc.cgi?acc=GSE61749.

## Additional file

Additional file 1:
**Supplementary figures and tables**. (PDF 320 kb)
