# Supplementary material for: Bacterial transcriptome reorganization in thermal adaptive evolution
Source: BMC Genomics. 2015 Oct 16;16:802. doi: 10.1186/s12864-015-1999-x (PMC4609109; doi:10.1186/s12864-015-1999-x)
Supplement: Additional file 1: — Supplementary figures and tables. (PDF 320 kb) [file 12864_2015_1999_MOESM1_ESM.pdf]

Bacterial transcriptome reorganization in thermal adaptive evolution

BW Ying *et al*

Supporting Information

Supplementary Figures and Figure Legends (Figures S1–S7) **p. 2-8**

Supplementary Tables (Tables S1–S2) **p. 9-10**

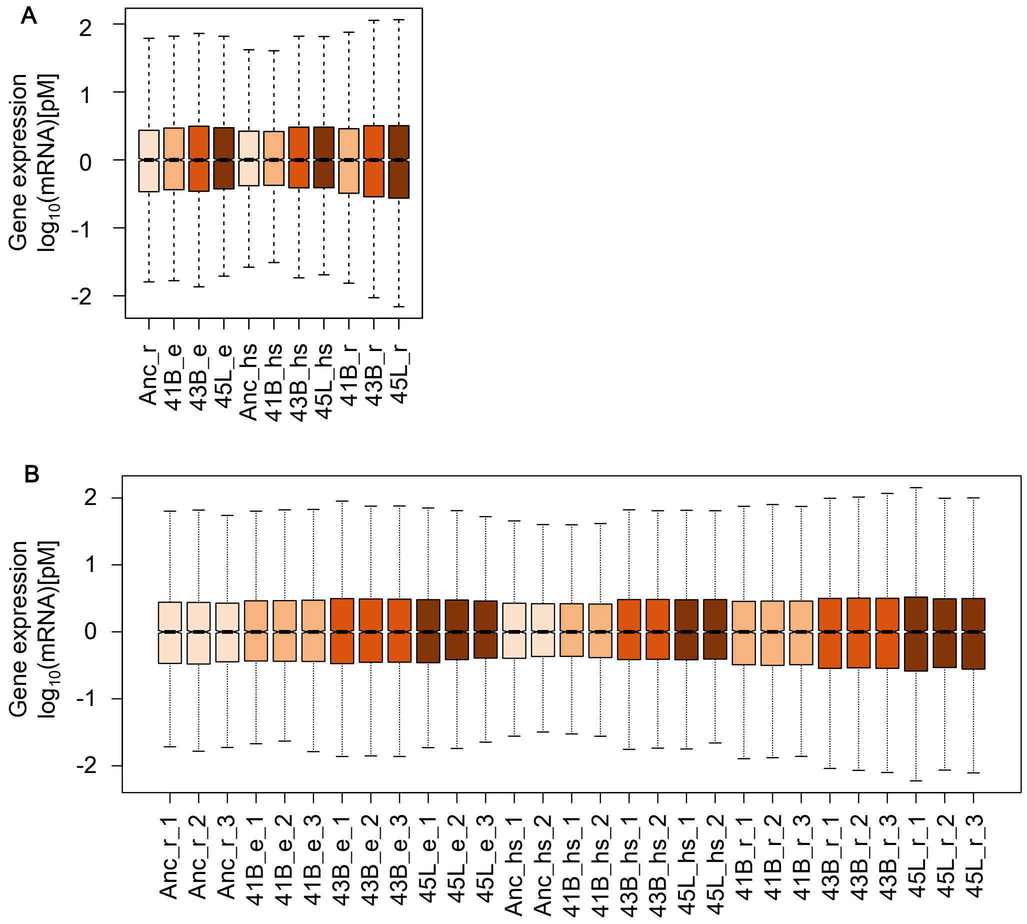

Figure S1 Box plots of gene expression. (A) Averaged expression data sets of each condition and (B) individual microarray tests. The color variation represents the difference in strains. The three experimental conditions of heat shock response, steady states at regular and evolutionary temperatures are indicated as hs, r and e, respectively. Repeated tests are marked with numbers. The gene expression levels are shown in log-scale mRNA concentration.

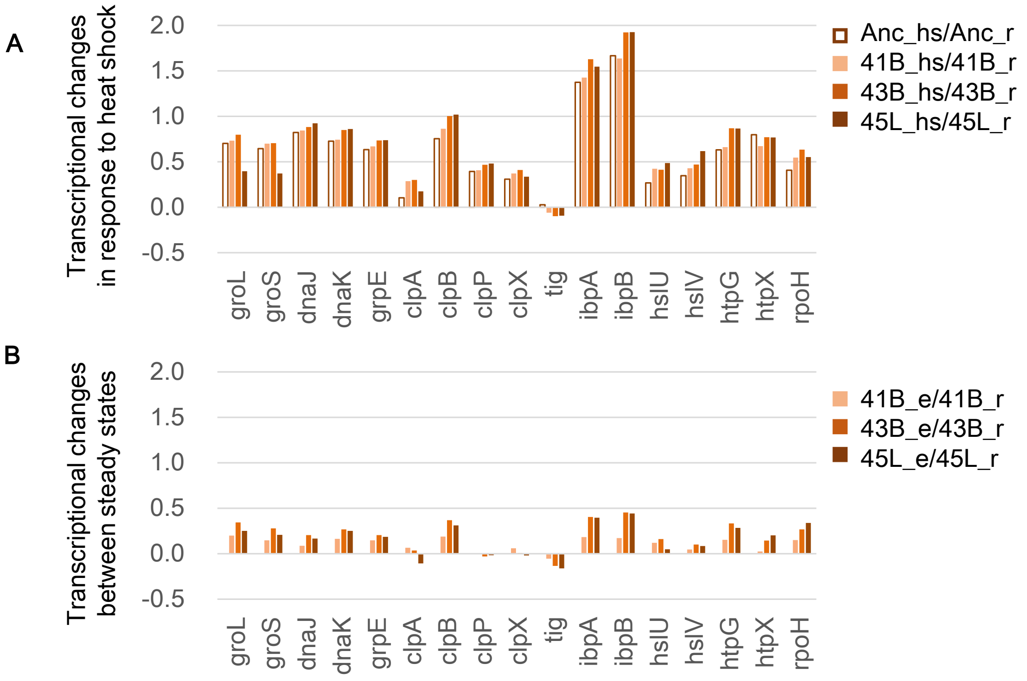

Figure S2 Changes in expression of the genes responsible for heat shock. A. Transcriptional changes in response to heat shock. The ratios of heat shock expression levels (_hs) to steady expression levels at regular temperature (_r) are shown. **B.** Transcriptional changes between two steady states. The ratios of steady expression at evolutionary temperature (_e) to that at the regular temperature (_r) are shown. The color variation represents the difference in strains as indicated. The genes with heat shock associated expression were selected based on previous literature (Mogk *et al*, 2001, *Molecular Chapreones in the Cell*, p.1-34, Oxford). The ratios are in log-scale unit.

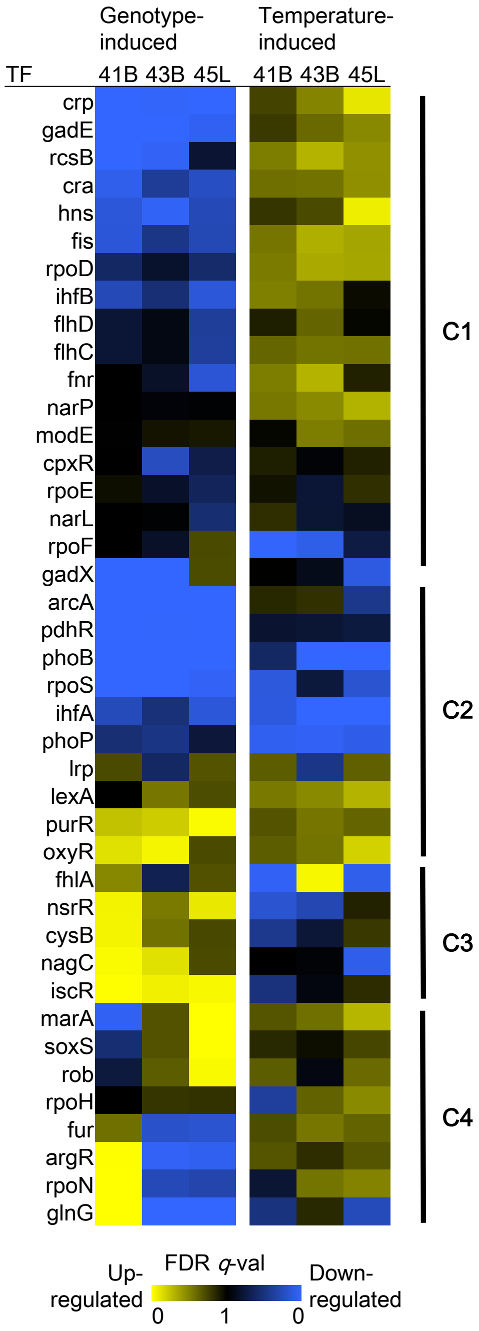

Figure S3 Enriched gene regulations. Heat maps of enriched gene links based on regulatory networks (TF). The left and right panels represent comparisons of genotypes and growth temperatures, respectively. The statistical significance (FDR *q* value) is indicated as the heat map. Vivid colors represent high significance in the directions of either up-regulated (yellow) or down-regulated (blue) genes. The strains are indicated. The numbered black bars located at the right of the heat maps stand for the roughly categorized clusters showing distinguished changing patterns, as described in Figure 3.

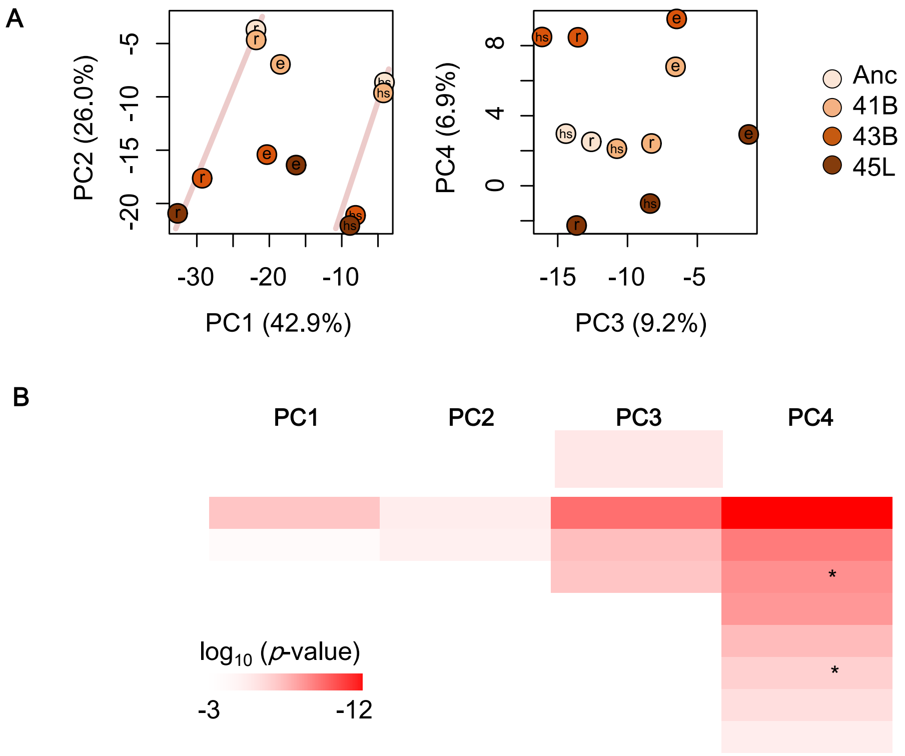

Figure S4 Principal component analysis. A. Principal component analysis based on averaged data sets. Because the first four PCs represented more than 70% of the entire data set, only these four main components (P1-P4) are shown. Color variation filled in the circles represents differences between the strains (Anc, 41B, 43B and 45L), as indicated. The letters hs, r, and e, which are indicated within the circles, represent the gene expression conditions for heat shock responsive states, steady states at regular and evolutionary (high) temperatures, respectively. The weights of each PC are indicated. Pale pink lines illustrate two zones of the steady expression at a regular temperature and the responsive expression, respectively. **B.** Gene categories and gene regulatory links significantly contributed to the main PCs. The top 5% of the weighted genes (total 439 genes) for each PC were subjected to analysis. Color bars indicate statistical significance in log-scaled *p* values obtained using binomial tests with Bonferroni corrections. Asterisks indicate either non-synonymous single-nucleotide substitutions or InDel mutations that occurred during evolution.

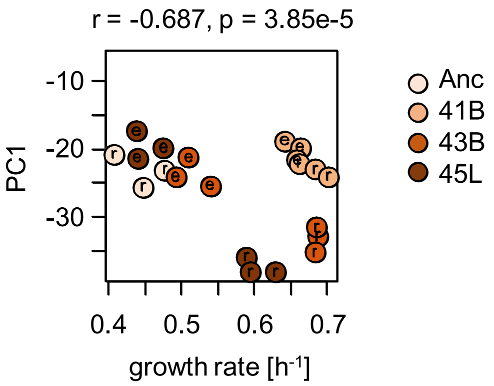

Figure S5 Correlation between PC1 and growth rate. The analysis was performed as described in Figure 4, based on gene expression levels. The relationship between PC1 and growth rate is shown. The correlation coefficient (r) and p values are indicated. Color variation is as described.

PC1	PC2	PC3	PC4
nac	nac	cysD	cysD
cysD	rpoD	ilvC	groL
ilvC	groL	lldP	gltA
yehB	ylcE	ydeO	lldP
ygfO	ydeO	yhiD	yhgF
tpx	oxyR	kduI	ylcE
groL		gltB	ydeO
pyrD			yhiD
rpoD			flu
lrp			oxyR
rho			gltB
ydeO			
pykF			
yhiD			
pykF			

Figure S6 Mutated genes in the four PCs. The mutated genes (Kishimoto *et al*, 2010) significantly weighted in the four PCs are listed. The gene names are indicated and the color variation among green, orange, and blue represents the mutation types, synonymous and nonsynonymous substitutions, and InDel (insertion or deletion) mutations, respectively.

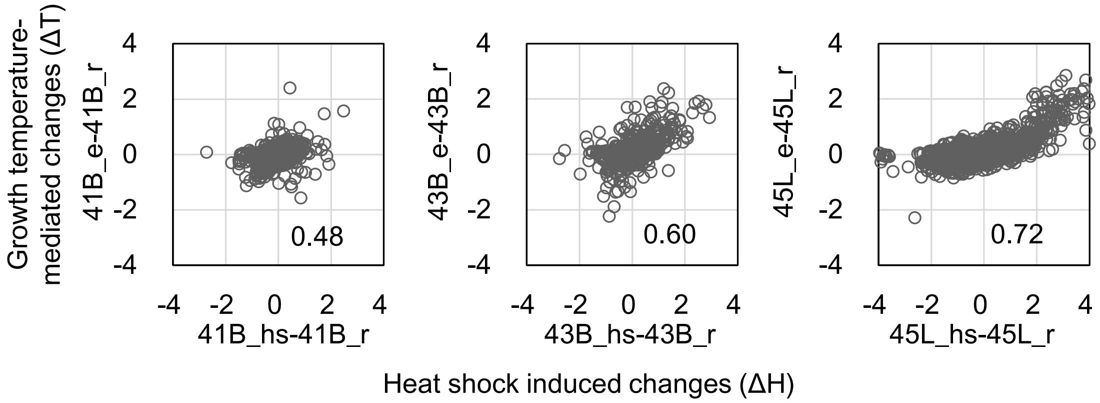

Figure S7 Correlations of transcriptional changes between the transient and the long-term temperature increase. Scatter plots of the changes in gene expression mediated by heat shock and growth temperatures. The heat shock-induced changes in gene expression (ΔH) are plotted against the growth temperature-mediated changes in gene expression (ΔT). Correlation coefficients are indicated.

Table S1 Transcriptional regulators carrying non-synonymous single-nucleotide substitution or InDel mutations. The mutated genes that are hypothesized to play a role in transcriptional regulation are summarized from previously reported mutations found during thermal adaptive evolution (Kishimoto et al, 2010).

Period	Gene name	Gene function
Anc→41B	*uhpA*	DNA-binding response regulator
	rpoD	RNA polymerase, sigma 70 (sigma D) factor
41B→43B	*lrp*	DNA-binding transcriptional dual regulator
	rho	transcription termination factor
43B→45L	*oxyR*	DNA-binding transcriptional dual regulator
	nadR	bifunctional DNA-binding transcriptional repressor
	putA	fused DNA-binding transcriptional regulator

Table S2 Epistasis in PCs 1-4. Analysis as described in Figure 5A was performed for the top 5% of genes that weighted individually and overlapped in PC1 to PC4. All unique and overlapping genes in PCs 1-4 totaled 4383 genes; the top 439 genes were weighted in PC1-4 independently; and 48 common genes were weighted in all PCs.

Strains	41B	43B	45L
All	23%	23%	32%
PC1 top 5%	17%	16%	34%
PC2 top 5%	25%	20%	32%
PC3 top 5%	24%	25%	37%
PC4 top 5%	27%	23%	38%
PCs 1–4 overlaps	28%	20%	50%
